# Vasoactive-Inotropic Score and Vasoactive-Ventilation-Renal Score as Outcome Predictors for Children on Extracorporeal Membrane Oxygenation

**DOI:** 10.3389/fped.2021.769932

**Published:** 2021-11-30

**Authors:** Ira Shukla, Sheila J. Hanson, Ke Yan, Jian Zhang

**Affiliations:** ^1^Section of Critical Care, Department of Pediatrics, Tulane University School of Medicine, New Orleans, LA, United States; ^2^Section of Critical Care, Department of Pediatrics, Medical College of Wisconsin, Milwaukee, WI, United States; ^3^Section of Quantitative Health Sciences, Department of Pediatrics, Medical College of Wisconsin, Milwaukee, WI, United States

**Keywords:** extracorporeal circulation, cardiovascular agents, child, infant, critical care outcomes, outcome assessment, health care

## Abstract

We aimed to determine the association of vasoactive-inotropic score (VIS) and vasoactive-ventilation-renal (VVR) score with in-hospital mortality and functional outcomes at discharge of children who receive ECMO. A sub-analysis of the multicenter, prospectively collected data by the Collaborative Pediatric Critical Care Research Network (CPCCRN) for Bleeding and Thrombosis on ECMO (BATE database) was conducted. Of the 514 patients who received ECMO across eight centers from December 2012 to February 2016, 421 were included in the analysis. Patients > 18 years of age, patients placed on ECMO directly from cardiopulmonary bypass or as an exit procedure, or patients with an invalid or missing VIS score were excluded. Higher VIS (OR = 1.008, 95% CI: 1.002–1.014, *p* = 0.011) and VVR (OR: 1.006, 95% CI: 1.001–1.012, *p* = 0.023) were associated with increased mortality. VIS was associated with worse Pediatric Cerebral Performance Category (PCPC) (OR = 1.027, 95% CI: 1.010–1.044, *p* = 0.002) and Pediatric Overall Performance Category (POPC) score (OR = 1.023, 95% CI: 1.009–1.038, *p* = 0.002) at discharge. No association was found between VIS or VVR and Functional Status Score (FSS) at discharge. Using multivariable analyses, controlling for ECMO mode, ECMO location, ECMO indication, primary diagnosis, and chronic diagnosis, extremely high VIS and VVR were still associated with increased mortality.

## Introduction

Extracorporeal membrane oxygenation (ECMO) is increasingly used in pediatric patients with life-threatening cardiac and respiratory disorders (1). In more recent years, it has also been incorporated and used as part of extracorporeal cardiopulmonary resuscitation (E-CPR). However, there are no standardized guidelines on when to initiate ECMO in order to optimize survival and functional outcomes.

According to the Extracorporeal Life Support Organization (ELSO) registry, there are two scores established to predict mortality for pediatric patients requiring ECMO for respiratory failure, the Pediatric Risk Estimation Score for Children Using Extracorporeal Respiratory Support (PED-RESCUERS) (2) and Pediatric Pulmonary Rescue with Extracorporeal Membrane Oxygenation Prediction Score (P-PREP) (3). These scores are developed and validated only for patients requiring ECMO for a primary respiratory indication and neither of them looked at functional status as their outcomes.

Other scores have been developed to predict mortality and functional outcome in the non-ECMO setting. The Vasoactive-Inotropic Score (VIS) has been validated to be used to predict outcomes such as mortality and need for mechanical circulatory support, renal failure, cardiac arrest after infant cardiac surgery (4–6) as well as sepsis (7). The Vasoactive-Ventilation-Renal (VVR) Score has been recently developed and subsequently validated in the pediatric post-operative cardiac surgical population to predict mortality (8, 9).

Our objective with this study was to evaluate the association of VIS and VVR scores immediately prior to ECMO initiation with in-hospital mortality and functional outcome at discharge, and thereby, be useful in research design risk-stratification and in counseling the families of these patients.

## Materials and Methods

Patients were selected through the de-identified Bleeding and Thrombosis during ECMO (BATE) database released for the public use by the Collaborative Pediatric Critical Care Research Network (CPCCRN) (10). To prepare the analytical database for the analysis of the current and future derivative studies, all patient identifiers were recoded, and a de-identified data set was created in accordance with definitions of the Health Insurance Portability and Accountability Act (HIPAA). Data was collected on all patients receiving ECMO at eight CPCCRN sites over a 21-month period from December 2012 to September 2014. Patient data on demographics, baseline labs, baseline functional status, VIS score, ventilation details, primary and secondary diagnosis, co-morbid conditions, mortality, hospital discharge data, ECMO information, and functional status at discharge were obtained from the database.

Secondary analysis was conducted after obtaining Children's Hospital of Wisconsin Institutional review board approval for non-human subject research. Data was received by the CPCCRN network in SAS format. Exclusion criteria were patients > 18 years of age at ECMO initiation, patients placed on ECMO directly from cardiopulmonary bypass or as an exit procedure, or patients with an invalid or missing VIS score. Frequencies and percentages were generated for categorical variables. Median and interquartile range (IQR) were summarized for continuous variables due to the skewness of the data. VIS score was already provided as part of the dataset.

### Equations for Score Calculations


$$\begin{array}{l} \text{ VVR = VIS + Ventilation index + Renal Score}\\ \text{VIS Score = Dopamine dose (mcg/kg/min)}\\ \;+\text{Dobutamine (mcg/kg/min) + 10}\\ \text{ × Milrinone dose (mcg/kg/min) + 10,000}\\ \text{ × Vasopressin dose (units/kg/min) + 100}\\ \text{ × Epinephrine dose (mcg/kg/min) + 100}\\ \text{ × Norepinephrine dose (mcg/kg/min)} \end{array}$$

Ventilation index = Ventilator RR × (PIP – PEEP) × PaCO2/1000; where RR is respiratory rate (breaths per minute), PIP is peak inspiratory pressure (cm H_2_O), PEEP is positive end-expiratory pressure (cm H_2_O), and PaCO2 is partial pressure of carbon dioxide in blood (mmHg).


$$\begin{array}{l} \text{Renal score = change in creatinine from post to}\\ \text{ pre − ECMO initiation × 10 } \end{array}$$

When the VVR score was first developed and subsequently validated in acyanotic pediatric patients who underwent cardiac surgery with cardio-pulmonary bypass (8, 9), the renal score was calculated using the difference between post-operative creatinine and the baseline creatinine prior to surgery. We used a modification of this by using the difference between the first post-cannulation creatinine and the most recent creatinine prior to ECMO cannulation.

### Functional Outcome Scores

Pediatric Overall Performance Category (POPC)score (11), Pediatric Cerebral Performance Category (PCPC) score (11), and the Functional Status Score (FSS) scales (12, 13). The POPC and PCPC scores were developed in 1992 for measurement of short-term physical and cognitive disability after critical illness, ranging from 1 (normal) to 6 (death). Since then, these have been validated (13) and used in research studies pertaining to outcomes. The FSS is a more recently developed score consisting of six domains: mental status, sensory, communication, motor function, feeding, and respiratory. Of the 836 patients studied by Pollack et al. (12), 18% of patients had an FSS of 6, while 6% had a score ≥ 20. The average score was 10.3 ± 4.4 and the highest recorded score was 29. This tool was found to correlate closely (13) with traditionally used POPC and PCPC scores but provides a more detailed assessment of the patient's overall functional status using adaptive behavior.

Logistic regression analysis was used for determining association with hospital mortality. For functional outcomes, analysis was performed using logistic regression with the outcome scores being categorized as unfavorable (POPC>3, PCPC > 3, FSS ≥ 18) vs. favorable.

For secondary outcomes hospital length of stay, ICU length of stay, and ventilator-free days, the Kruskal–Wallis test and Mann–Whiney test were used to compare different categories of predictor variables.

Multivariable logistic regression analysis was performed to evaluate the association of VIS or VVR on hospital mortality after controlling for ECMO mode, ECMO location, ECMO indication, and primary and chronic diagnosis. These covariates were entered into the model one at a time, because they were highly correlated. To show the association of VIS or VVR with mortality after consideration of possible interactions between other predictors, a classification tree analysis was performed. This is a nonparametric recursive classification method that can identify interactions and possible thresholds without limiting input variables. The tree was optimized with the Gini method and 10-fold cross validation. The split criteria minimum was 10 for the parent nodes and 5 for the terminal nodes. For each predictor, the tree produced a variable importance score that ranged from 0 (not important) to 100 (very important), reflecting its placement and frequency of appearance in the tree. Variables with high importance score may not necessarily appear in the tree if they are acting as surrogate splitters behind the scenes. Variables exhibited in the tree or having a variable importance score ≥20 were reported. The Chi-square test or Fisher's exact test was used to generate the *p*-values for tree splits. No multivariable analysis was performed for outcomes PCPC, POPC, and FSS scores, due to the limited number of patients with unfavorable outcomes.

SAS 9.4 and SPM 8.2 were used for the analyses. A *p* <0.05 was considered as statistically significant.

## Results

Of the 514 patients in the BATE database, 93 were excluded: patients > 18 years of age at ECMO initiation (7), placed on ECMO directly from cardiopulmonary bypass or as an exit procedure (10), and patients with an invalid (2) or missing (74) VIS score. As shown in Table 1, of the 421 patients that were included, 53% were neonates, and 24% were ages 1 month to 23 months old. The most common primary diagnosis was respiratory in 212 (50%) patients, followed by cardiac in 144 (34%) patients. The mode of the cannulation was veno-arterial (VA) in 345 (83%) while the rest were veno-venous (VV). The most common primary diagnosis in patients requiring VV ECMO was respiratory distress/failure (85%). Three patients had cardiovascular disease—congenital as the primary diagnosis, and 1 patient in each of the following primary diagnoses: cardiac arrest sepsis/SIRS/septic shock, gastrointestinal disorder, drowning/asphyxia/hanging, congenital anomaly or chromosomal defect, cancer, and hematologic disorder. However, the indication for ECMO for all patients requiring VV ECMO was respiratory. The most common primary diagnosis in patients requiring VA ECMO was also respiratory distress/failure (42%) followed by cardiovascular disease—congenital (34%). The most common indication for ECMO in these cases was respiratory (43%), followed by cardiac (38%) followed by ECPR (19%). The median VIS score prior to ECMO cannulation was 15 (IQR 5-30), and the median VVR score was 50 (IQR 31-75). There were 236 (56%) patients alive at discharge. For survivors, the median hospital length of stay was 52 days (IQR 31-81) and median ICU length of stay was 36 days (IQR 22-69). The median duration of ECMO was 5 days (IQR 3-9). The median POPC score at discharge for survivors was 2 (IQR 2-3) and median PCPC was 2 (IQR 1-2).

**Table 1:** Characteristics of Patients and Extracorporeal Membrane Oxygenation (ECMO).

**Characteristic**	**N (%) or Median (IQR)**
**Patient Characteristic**	
Age	
- <1mo	225 (53)
- 1mo– 23 mo	101 (24)
- 2–5 yrs	36 (9)
- 6–12 yrs	26 (6)
- 13–18 yrs	33 (8)
Sex	
- Male	245 (58)
- Female	176 (42)
Race	
- White	204 (48)
- Black or African American	73 (17)
- Asian	15 (4)
- American Indian or Alaska Native	5 (1)
- Unknown or Not Reported	124 (29)
Primary Diagnosis	
- Respiratory	212 (50)
- Cardiac	144 (34)
- Sepsis	17 (4)
- Neurologic	3 (1)
- Other	45 (11)
VIS^1^ Score	15 (5–30)
VVR^2^ score	50 (30–75)
Lactate (mmol/L)	0.6 (0.2–1.7)
**ECMO Characteristic**	
ECMO indication	
- Respiratory	227 (54)
- Cardiac	129 (31)
- ECPR	65 (15)
Mode of ECMO	
- Veno-arterial (VA)	345 (83)
- Veno-venous (VV)	76 (17)
Location of ECMO care	
- PICU	90 (22)
- NICU	153 (36)
- CICU	178 (42)
**Outcomes^a^**	
Mortality	185(44)
PCPC at discharge	2 (1–2)
POPC at discharge	2 (2–3)
FSS at discharge	8 (7–10)
ECMO duration in days	5 (3–9)
Hospital LOS in days	52 (31–81)
ICU LOS in days	36 (22–69)
Ventilator-free days	23 (19–25)

*Mo, months; yrs, years; ECPR, Extracorporeal Cardiopulmonary resuscitation; PICU, Pediatric intensive care unit; NICU, Neonatal intensive care unit; CICU, Cardiac intensive care unit; VIS, Vasoactive-inotropic score; VVR, Vasoactive-ventilation-renal score; mmol/L, millimoles per liter; PCPC, Pediatric Cerebral Performance Category; POPC, Pediatric Overall Performance Category; FSS, Functional Status Score; LOS, Length of stay; ICU LOS, Intensive Care Unit Length of stay*.

a *= outcomes only in survivors*.

*Formula*.

1 *= VIS Score = Dopamine dose(mcg/kg/min) + Dobutamine(mcg/kg/min) + 10 x Milrinone dose (mcg/kg/min) + 10,000 x Vasopressin dose(units/kg/min) + 100 x Epinephrine dose (mcg/kg/min) + 100 x Norepinephrine dose(mcg/kg/min)*.

2 *= VVR score = VIS + Ventilation Index + Renal score*.

*Ventilation index = Ventilator Respiratory rate in breaths per minute x (Peak Inspiratory Pressure in centimeters of water pressure – Positive End Expiratory Pressure in centimeters of water pressure) x Partial pressure of Carbon dioxide in blood in millimeters of mercury/1000*.

*Renal score = change in creatinine in milligrams per deciliters x 10*.

### Univariable Analysis for Association With Mortality

As shown in Table 2, age, sex, and race were not significantly associated with hospital mortality. Higher VIS and higher VVR were associated with hospital mortality (OR = 1.008, 95% CI:1.002–1.014, *p* = 0.011, and OR = 1.006, 95% CI: 1.001–1.012, *p* = 0.023, respectively) when analyzed as a continuous variable but were not significantly associated with mortality as categorical predictor variables.

**Table 2:** Association of predictor variables with mortality by univariable analysis.

**Variable**	**OR (95% CI)**	***P*-value**
Age		
- <1mo	Reference	
- 1mo- 23mo	1.36 (0.85–2.18)	
- 2-5 years	1.50 (0.74–3.04)	0.27
- 6-12 yrs	0.94 (0.41–2.16)	
- 13-18 yrs	2.04 (0.97–4.27)	
Sex		
- Male	Reference	0.74
- Female	1.07 (0.72–1.58)	
Race		
- White	Reference	
- African American	1.03 (0.60–1.76)	0.60
- Other	1.61 (0.64–4.06)	
Primary diagnosis		
- Cardiac	1.89 (1.23–2.91)	0.0034
- Other	2.09 (1.19–3.67)	
- Respiratory	Reference	
Chronic diagnosis		
- Yes	1.63 (1.09–2.43)	
- No	Reference	0.018
ECMO indication		
- Cardiac	1.81 (1.17–2.81)	
- ECPR	(1.77–5.57)	
- Respiratory	Reference	0.0001
ECMO mode		
- VA	2.20 (1.28–3.77)	0.0042
- VV	Reference	
ECMO Location		
- CICU	1.82 (1.17–2.84)	0.029
- PICU	1.43 (0.84–2.42)	
- NICU	Reference	
VIS Score	1.008 (1.002–1.014)	0.011
VVR score	1.006 (1.001–1.012)	0.023
Lactate (mmol/L)	1.002 (0.995–1.008)	0.66

*ECMO, Extracorporeal Membrane oxygenation; ECPR, Extracorporeal Cardiopulmonary resuscitation; VA, Veno-arterial; VV, Veno-venous; VIS, Vasoactive inotropic score; VVR, Vasoactive- ventilation-renal score; PICU, Pediatric intensive care unit; NICU, Neonatal intensive care unit; CICU, Cardiac intensive care unit; mmol/L, millimoles per liter*.

### Univariable Analysis for Association With Functional Outcomes in Survivors

Twenty of the survivors had unfavorable functional outcome with POPC >3, 11 with PCPC >3 and 6 with FSS >18. With analysis of the functional outcomes of PCPC, POPC, and FSS scores by favorable and unfavorable categories (Table 3), VIS was significantly associated with unfavorable POPC (OR = 1.023; 95% CI:1.009–1.038, *p* = 0.0019) and PCPC (OR = 1.027; 95% CI: 1.010–1.044, *p* = 0.0015). None of the predictor variables was associated with FSS as a categorical outcome.

**Table 3:** Logistic regression of association of Predictor Variables with an unfavorable functional outcome in survivors.

**Categorical outcome**	**POPC** **>3**		**PCPC** **>3**		**FSS** **≥** **18**	
	**OR (CI)**	***P*-value**	**OR (CI)**	***P*-value**	**OR (CI)**	***P*-value**
ECMO Mode						
- VA	Reference	0.81	Reference	0.72	Reference	0.54
- VV	1.14 (0.39–3.28)		1.28 (0.33–5.00)		1.71 (0.31–9.61)	
ECMO Location						NS
- PICU	1.44 (0.37–5.64)	0.41	5.55 (0.56–54.87)	0.21	0.58 (0.06–5.71)	0.81
- NICU	2.10 (0.70–6.30)		6.69 (0.81–55.51)		0.59 (0.10–3.62)	
- CICU	Reference		Reference		Reference	
ECMO Indication						
- Respiratory	8.93 (1.17–68.31)	0.067	n/a		n/a	
- ECPR	2.78 (0.17–46.29)					
- Cardiac	Reference					
Chronic diagnosis						
- Yes	1.46 (0.56–3.80)	0.44	1.35 (0.38–4.74)	0.64	0.76 (0.15–3.82)	0.73
- No	Reference		Reference		Reference	
VIS score	1.023 (1.009–1.038)	0.002	1.027 (1.010–1.044)	0.002	0.99 (0.95–1.03)	0.62
VVR score	1.011 (0.994–1.027)	0.20	1.022 (0.999–1.045)	0.059	0.96 (0.88–1.04)	0.27

*n/a, Unable to generate due to limited numbers*.

*ECMO, Extracorporeal Membrane oxygenation; ECPR, Extracorporeal Cardiopulmonary resuscitation; VA, Veno-arterial; VV, Veno-venous; VIS, Vasoactive inotropic score; VVR, Vasoactive- ventilation-renal score; PICU, Pediatric intensive care unit; NICU, Neonatal intensive care unit; CICU, Cardiac intensive care unit; POPC, Pediatric overall performance category at discharge; PCPC, Pediatric cerebral performance category at discharge; FSS, Functional status scale at discharge; OR, Odds ratio; CI, Confidence interval*.

### Bivariable Analysis

When sequentially adjusted for ECMO mode, ECMO location, ECMO indication, primary diagnosis, and chronic diagnosis, VIS and VVR are still significantly associated with mortality (Table 4).

**Table 4:** Bivariable analysis of association of vasoactive-inotropic score (VIS) and vasoactive-ventilation-renal score (VVR) with mortality.

**Variable adjusted**	**VIS OR (95% CI); *P*-value**	**VVR OR (95% CI); *P*-value**
Primary diagnosis	1.008 (1.001–1.014); 0.016	1.008 (1.002–1.015); 0.009
Chronic Diagnosis	1.008 (1.002–1.014); 0.0098	1.007 (1.001–1.013); 0.016
ECMO mode	1.007 (1.001–1.013); 0.016	1.007 (1.001–1.013); 0.021
ECMO location	1.008 (1.002–1.014); 0.0097	1.008 (1.002–1.014); 0.0083
ECMO indication	1.009 (1.002–1.015); 0.0087	1.008 (1.002–1.014); 0.010

*ECMO, Extracorporeal Membrane oxygenation; VIS, Vasoactive inotropic score; VVR, Vasoactive- ventilation-renal score; OR, Odds ratio; CI, Confidence interval*.

### Multivariable Analysis

**Figure 1:**
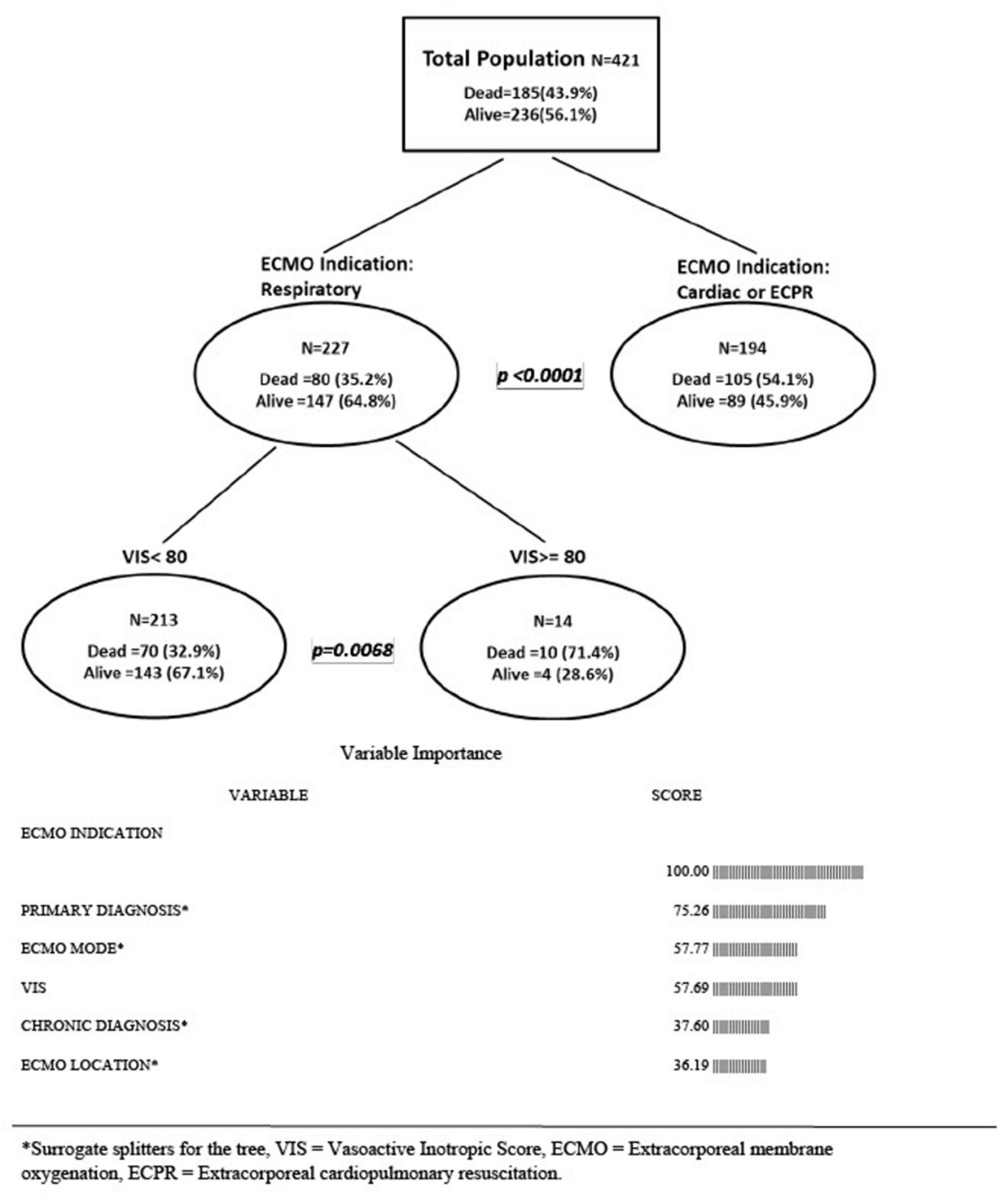
Multivariable classification tree analysis-association of vasoactive inotrope score and other risk factor with mortality. *Surrogate splitters for the tree, VIS, vasoactive inotropic score; ECMO, extracorporeal membrane oxygenation; ECPR, extracorporeal cardiopulmonary resuscitation.

Due to high correlation of the covariates, a multivariable classification tree analysis was performed to show the association of VIS or VVR with mortality after consideration of possible interactions between other predictors. As shown in Figure 1, when VIS was in the tree the most important predictor of mortality was ECMO indication illustrated by being the first branch point in the tree. Mortality for respiratory ECMO indication was 35.2 vs. 54.1% for cardiac/ECPR indications (*p* < 0.0001). For those with respiratory ECMO indication, extremely high VIS score at the time of ECMO initiation was a predictor of mortality. The branchpoint occurred at very high VIS scores; If VIS < 80, then the mortality was 32.9%; if VIS ≥ 80, then the mortality was 71.4% (*p* = 0.0068). (See Figure 1 for variable importance scores for when VIS was in the tree).

**Figure 2:**
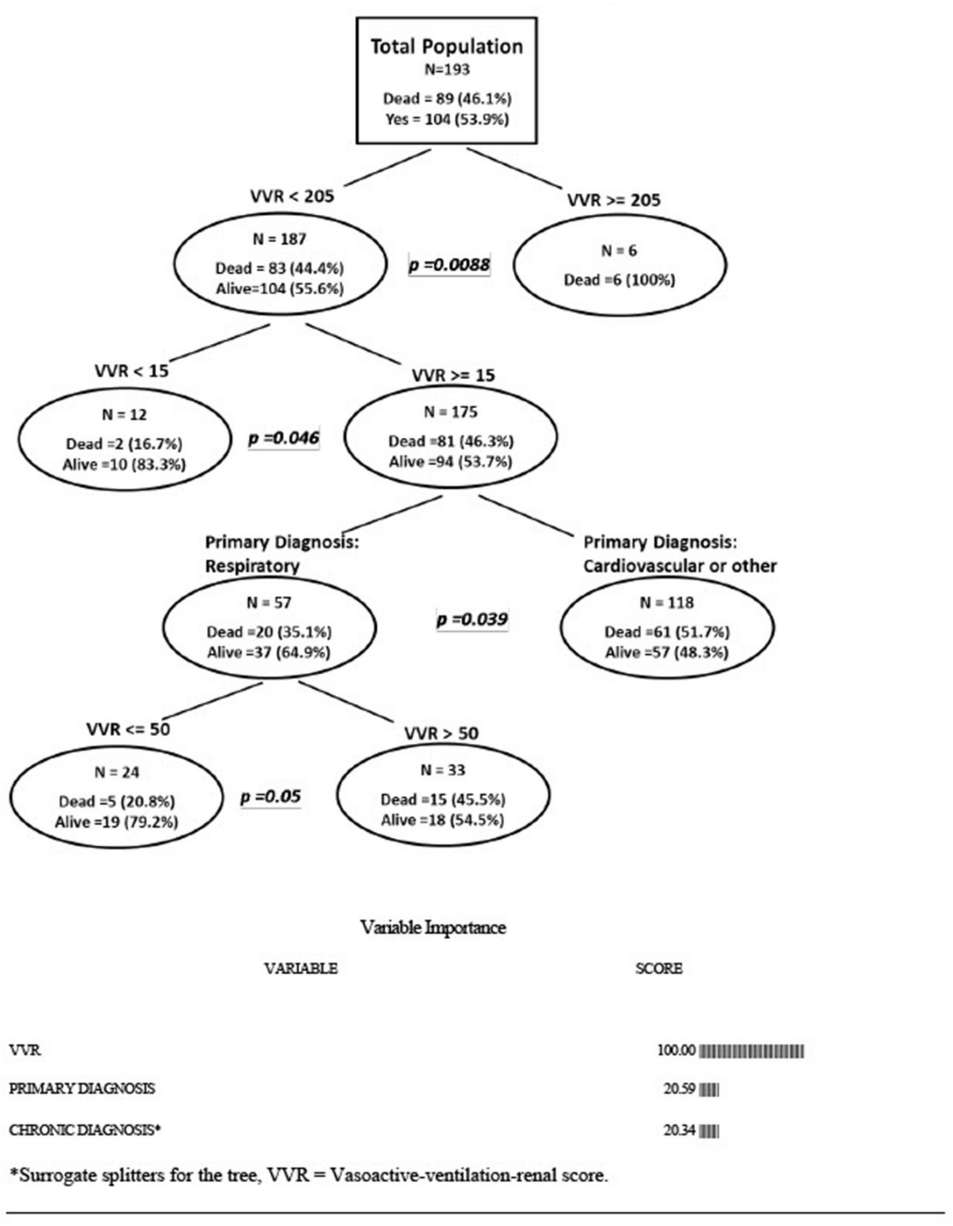
Multivariable classification tree analysis-association of vasoactive-ventilation-renal score and other risk factors with mortality. *Surrogate splitters for the tree; VVR, vasoactive-ventilation-renal score.

Analysis with VVR in the classification tree found the most important predictor of mortality to be VVR. If VVR < 205 then the mortality was 44.4%. If VVR ≥ 205, then the mortality was 100% (*p* = 0.0088). If VVR < 15, then the mortality was 16.7%; if VVR 15-205, then the mortality was 46.3%(*p* = 0.046). For those with VVR 15–205, the primary diagnosis was independently associated with mortality. For patients with respiratory diagnosis, the mortality was 35.1 vs. 51.7% for cardiac or other (*p* = 0.039). Following the analytic branchpoints, for those with VVR between 15 and 205 and a primary diagnosis of respiratory, if VVR ≤ 50, then the mortality was 20.8%. If VVR > 50, then the mortality was 45.5% (*p* = 0.05). (See Figure 2 for variable importance scores for when VVR was in the tree).

No multivariable analysis was done for outcomes PCPC, POPC, and FSS scores, due to the limited number of patients with unfavorable outcomes.

### Analyses of Secondary Outcomes for Survivors Only

No significant difference was found between VIS and VVR score with hospital length of stay or ICU length of stay. Survivors with a lower VVR score category were found to have fewer ventilator free days than those with a higher VVR score category (Table 5).

**Table 5:** Association of predictor variables with Hospital length of stay (LOS), Intensive care unit length of stay (LOS) and Ventilator free days.

**Parameter**	**Hospital LOS in days**	**Significance of association with hospital LOS in days**	**ICU LOS in days**	**Significance of association with ICU LOS in days**	**Ventilator free days**	**Significance of association with ventilator free days**
	**Median (IQR)**	***P* value**	**Median (IQR)**	**P value**	**Median (IQR)**	**P value**
ECMO Mode						
- VA	55 (33–84)	0.0022	37 (22–72)	0.13	23 (19–25)	0.018
- VV	36 (28–60)		32 (21–46)		22 (18–23)	
ECMO Location						
- PICU	54 (31–89)		42 (21–71)		23 (16–24)	
- CICU	54 (33–88)	0.22	43 (29–70)	0.017	25 (23–26)	<0.0001
- NICU	44 (30–76)		30 (19–47)		20 (17–23)	
ECMO Indication						
- Cardiac	56 (30–85)		35 (20–65)		24 (22–25)	
- Respiratory	50 (31–77)	0.20	38 (23–68)	0.83	21 (17–23)	<0.0001
- ECPR	53 (39–137)		35 (24–85)		25 (23–26)	
Primary diagnosis						
- Cardiac	57 (33–107)		35 (23–71)		25 (23–26)	
- Respiratory	50 (30–77)	0.15	37 (24–70)	0.31	20 (17–23)	<0.0001
- Other	51 (29–84)		31 (18–47)		23 (19–25)	
Chronic diagnosis						
- Yes	59 (33–95)	0.0011	39 (23–76)	0.03	23 (20–25)	0.0003
- No	44 (28–68)		33 (20–50)		21 (18–23)	
VIS score						
- <5	53 (35–107)		36 (18–58)		23 (19–25)	
−5–9	59 (45–77)		44 (32–73)		20 (16–25)	
−10–14	44 (28–61)	0.064	31 (23–5250	0.10	23 (19–25)	0.46
−15–19	67 (29–107)		(28–98)		21 (18–24)	
- >/= 20	42 (28–79)		35 (19–66)		23 (20–24)	
VVR score						
- <10	61 (50–74)		48 (38–53)		22 (19–23)	
−11–20	60 (43–112)		30 (18–54)		23 (21–23)	
−21–30	62 (40–90)	0.21	33 (27–39)	0.51	25 (24–26)	0.03
−31–40	37 (23–81)		32 (17–71)		25 (20–26)	
- >40	43 (30–85)		32 (20–54)		24 (20–25)	

*ECMO, Extracorporeal Membrane oxygenation; ECPR, Extracorporeal Cardiopulmonary resuscitation; NS, not significant; VA, Venoarterial; VV, Venovenous; PICU, Pediatric intensive care unit; NICU, Neonatal intensive care unit; CICU, Cardiac intensive care unit; VIS, Vasoactive-inotropic score; VVR, Vasoactive-ventilation-renal score; IQR, Interquartile range*.

## Discussion

As the use of ECMO in the pediatric population increases every year, the survival rate on ECMO does not show the same rate of increase (1). Despite advances in ECMO management strategies, there is no consensus on when to initiate ECMO and what risk factors affect outcomes. We analyzed the association of new risk scores, VIS and VVR, on mortality and functional outcome in children receiving ECMO.

In bivariable analysis, our study found that higher VIS or VVR score at initiation of ECMO is associated with higher in-hospital mortality when controlled sequentially for diagnosis, chronic diagnoses, ECMO mode, location, and indication. Multivariable analysis with classification tree analysis found VIS score was associated with mortality in patients going on ECMO for respiratory indications with heroically high VIS >80. The VIS score has been studied extensively and is a validated score in patients with sepsis and in post-cardiac bypass population to predict mortality. In 2014, Gaies et al. demonstrated that a VIS score > 20 in the first 24 h after infant cardiac surgery was associated with increased mortality after surgery (4).

A recent study by Cashen et al. (14), using the same BATE database as our study, did not demonstrate any association of VIS with mortality. We believe that this difference in results was because they categorized VIS into <20 and >20. We replicated these results when categorizing VIS into similar groups; however, when considering VIS as a continuous variable there was association with mortality in the univariable analysis with an odds ratio of 1.008 (95% CI is 1.002–1.014). This association with mortality persisted in the multivariable classification tree analysis for patients receiving ECMO for respiratory indications, with extremely high VIS >80 having a mortality of 71 vs. 33% for those with lower VIS at a time of cannulation for respiratory indications (Figure 1). The branch point of significant difference in mortality association occurs at a VIS of 80 which could be considered “heroic” or near code dosing compared to median VIS scores reported in this and other studies (4, 15). The hemodynamic instability requiring vasopressor support in severe respiratory failure is due in part from the unfavorable cardiopulmonary interactions resulting in right heart dysfunction from high mean airway ventilatory pressures needed prior to ECMO cannulation. ECMO offloads the right heart by allowing lower ventilatory support as the circuit oxygenates, removes carbon dioxide and corrects acidosis, effectively reversing the hemodynamic dysfunction secondary to significant mechanical ventilation. The clinical significance of a mortality association at extremely high degree of vasopressor therapy at the time of cannulation for respiratory indications may reflect a pre-morbid multiorgan failure rather than isolated cardiac dysfunction from unfavorable cardiopulmonary interactions.

Logistic regression analysis of survivors found only VIS score to be associated with unfavorable functional outcome by POCP or PCPC. Other predictor variables such as ECMO mode, location, and indication were not associated with unfavorable functional outcome. None of the predictor variables were associated with FSS as a functional outcome. Of the 236 survivors to hospital discharge, only 20 had poor functional outcome, limiting the power of this study.

We have been the first to use VVR score outside of the post-cardiac surgical population and found that, when analyzing VVR score at the time of ECMO cannulation as a continuous variable, it was associated with higher mortality. VVR considers respiratory, renal as well as cardiac dysfunction. VVR score was first developed by Miletic et al. (8) and demonstrated superiority to post-operative VIS score and lactate in predicting outcomes after pediatric cardiac surgery. The peak VVR score in the Miletic study was a median of 38.3 (IQR 12.5–142.9). These results were later validated in both single center (16) and multicenter (17) studies. In our study patients with a primary respiratory diagnosis, a VVR score of > 50 was associated with higher mortality than those patients with VVR ≤ 50 at the time of ECMO initiation (Figure 2). In general, extremely high VVR was associated with decreased survival to discharge.

The ELSO registry describes two scores, PED-RESCUERS (2) and P-PREP (3), that can be used as a tool to predict mortality in the Pediatric Respiratory ECMO. However, these scores are applied to patients receiving ECMO solely for respiratory indications. The only other study that developed and studied a prediction model for all indications of ECMO was recently published by Bailey et al. (18), who developed a more comprehensive Pediatric ECMO prediction model for predicting in-hospital mortality among children receiving ECMO support for any indication. Although they also used the data from the BATE dataset in their study, it did not include the VIS and VVR scores as part of the prediction model.

With this study we have been one of the few to examine the association of VIS or VVR scores with the short-term functional status at discharge of a patient who was on ECMO for any indication during their hospital stay. There have been several studies that have looked at short and long-term neuropsychological outcomes after ECMO in the neonatal and pediatric population (19–24) but there is lack of consensus in terms of useful predictors for functional outcomes. The more recent study by Cashen et al. (14) which also utilized the same BATE database as our study showed the VIS score was not significantly associated with a functional status score. This was replicated in our study as well. However, we did find a significant association between a higher VIS score associated with an increased likelihood of unfavorable POPC and PCPC score at hospital discharge. One of the reasons for this finding could be that POPC and PCPC scores are less objective than the FSS score (13). POPC and PCPC rely more heavily on clinical judgement for their calculation whereas FSS has categories more easily determined from the medical record to determine the functional status of the patient. There was no association between VVR score at ECMO initiation and PCPC, POPC, or FSS score at discharge.

We did identify some limitations in our study. One limitation is that the baseline functional scores were recorded as the patient's functional status on the day of ECMO initiation and not a baseline functional status of the patient prior to admission. This is the reason why we decided to study the association of predictor variables with only the functional outcome scores at discharge rather than the change in functional status from baseline to discharge.

The design of the original BATE study was such that baseline values were collected on the day of ECMO initiation. Due to this reason, we had the creatinine measurements from no earlier than the day of ECMO initiation. As mentioned earlier, the prior studies that developed and validated the VVR score had used the difference between post-surgical creatinine and baseline creatinine (8, 9). Given the design of the database, the baseline creatinine for our study was available very close to the date and time of ECMO initiation, if not on the day of cannulation in most cases. This may have resulted in the baseline creatinine level to be higher than the patient's healthy baseline. When using this baseline creatinine on the day of cannulation for calculation of renal score, which is part of the VVR score, we may have underestimated the association of renal injury in predicting mortality.

Bedside tools to assess prognosis of outcome can assist in a clinician's decision making as to the timing and initiation of ECMO. They are also useful for risk classification in research endeavors to evaluate the effect of interventions on ECMO outcomes. Adding VIS and VVR to this risk stratification is appropriate. Future work, in a larger population, is needed to fully evaluate the association of VIS and VVR score at time of ECMO initiation with functional outcome of survivors.

## Conclusion

Extremely high VIS and VVR score at the time of ECMO initiation is associated with higher in-hospital mortality and a higher VIS score is associated with an unfavorable POPC and PCPC score in ECMO survivors. These risk factors can be another tool to guide clinician decision making regarding initiation of ECMO and to risk stratify patients in future research studies.

## Data Availability Statement

Dataset available via the Collaborative Pediatric Critical Care Research Network by application. Requests to access these datasets should be directed to Stephanie Dorton, stephanie.dorton@hsc.utah.edu.

## Author Contributions

SH: faculty mentor for this project and helped with the initial idea and execution and editing manuscript. IS: initial idea, literature search, and writing manuscript and figures and tables. KY and JZ: biostatistician, helped in analysis and interpretation, edited the manuscript, figures, and tables. All authors contributed to the article and approved the submitted version.

## Conflict of Interest

The authors declare that the research was conducted in the absence of any commercial or financial relationships that could be construed as a potential conflict of interest.

## Publisher's Note

All claims expressed in this article are solely those of the authors and do not necessarily represent those of their affiliated organizations, or those of the publisher, the editors and the reviewers. Any product that may be evaluated in this article, or claim that may be made by its manufacturer, is not guaranteed or endorsed by the publisher.
